# Crystal structure of bis­{1-[(*E*)-(2-meth­oxy­phen­yl)diazen­yl]naphthalen-2-olato-κ^3^ *O*,*N* ^2^,*O*′}copper(II) containing an unknown solvate

**DOI:** 10.1107/S2056989015019817

**Published:** 2015-10-31

**Authors:** Souheyla Chetioui, Noudjoud Hamdouni, Djamil-Azzeddine Rouag, Salah Eddine Bouaoud, Hocine Merazig

**Affiliations:** a Unité de Recherche de Chimie de l’Environnement et Moléculaire Structurale (CHEMS), Faculté des Sciences Exactes, Département de Chimie, Université Constantine 1, Constantine 25000, Algeria; b Laboratoire de Cristallographie, Département de Physique, Université Constantine 1, Constantine 25000, Algeria

**Keywords:** crystal structure, naphthalen-2-olate, copper(II) complex, octa­hedral coordination, azo compounds

## Abstract

The title complex, [Cu(C_17_H_13_N_2_O_2_)_2_], crystallizes with two independent mol­ecules in the asymmetric unit. Each Cu^II^ atom has a distorted ocahedral coordination environment defined by two N atoms and four O atoms from two tridentate 1-[(*E*)-(2-meth­oxy­phen­yl)diazen­yl]naphthalen-2-olate ligands. In the crystal, the two mol­ecules are linked *via* weak C—H⋯O hydrogen bonds which in turn stack parallel to [010]. A region of disordered electron density, most probably disordered methanol solvent molecules, was corrected for using the SQUEEZE routine in *PLATON* [Spek (2015). *Acta Cryst.* C**71**, 9–18]. Their formula mass and unit-cell characteristics were not taken into account during refinement.

## Related literature   

For applications of azo compounds, see: Millington *et al.* (2007 ▸); Hallas & Choi (1999 ▸); Ho *et al.* (1995 ▸); Sharma *et al.* (2008 ▸). For related structures, see: Tai *et al.* (2010 ▸); Lin *et al.* (2010 ▸).
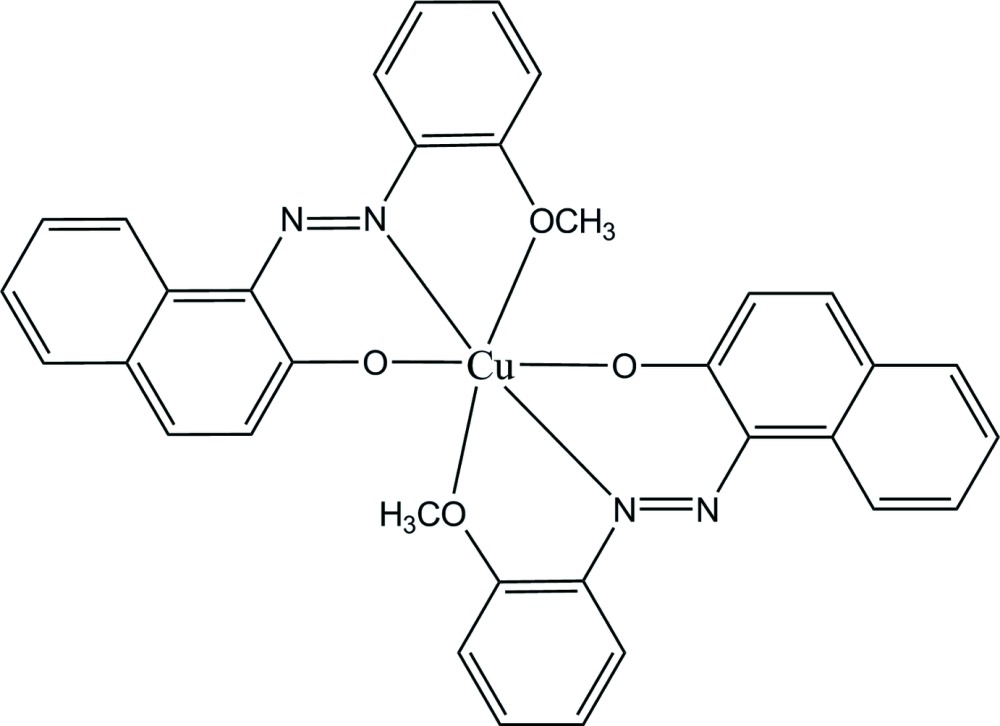


## Experimental   

### Crystal data  

[Cu(C_17_H_13_N_2_O_2_)_2_]
*M* *_r_* = 618.13
Monoclinic 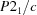

*a* = 29.749 (2) Å
*b* = 12.5171 (9) Å
*c* = 15.3565 (12) Å
β = 92.969 (5)°
*V* = 5710.7 (8) Å^3^
*Z* = 8
Mo *K*α radiation
μ = 0.81 mm^−1^
*T* = 100 K
0.02 × 0.01 × 0.01 mm

### Data collection  

Bruker APEXII diffractometer
76488 measured reflections
20125 independent reflections
9009 reflections with *I* > 2σ(*I*)
*R*_int_ = 0.122

### Refinement  

*R*[*F* ^2^ > 2σ(*F* ^2^)] = 0.074
*wR*(*F* ^2^) = 0.168
*S* = 1.01
20125 reflections
775 parameters
H-atom parameters constrained
Δρ_max_ = 0.55 e Å^−3^
Δρ_min_ = −1.01 e Å^−3^

### 

Data collection: *APEX2* (Bruker, 2006 ▸); cell refinement: *APEX2*; data reduction: *APEX2*; program(s) used to solve structure: *SIR92* (Giacovazzo *et al.*, 1992 ▸); program(s) used to refine structure: *SHELXL2014* (Sheldrick, 2015 ▸); molecular graphics: *ORTEP-3 for Windows* (Farrugia, 2012 ▸); software used to prepare material for publication: *WinGX* (Farrugia, 2012 ▸) and SQUEEZE in *PLATON* (Spek, 2015 ▸).

## Supplementary Material

Crystal structure: contains datablock(s) Chetioui_enrt_0m, I. DOI: 10.1107/S2056989015019817/cq2017sup1.cif

Structure factors: contains datablock(s) I. DOI: 10.1107/S2056989015019817/cq2017Isup2.hkl

**Fig. 1.**
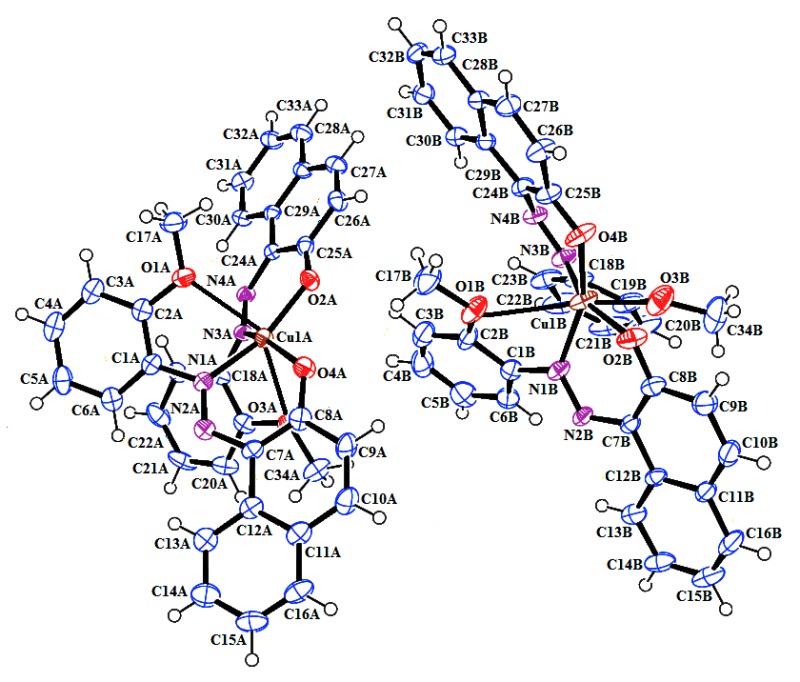
The molecular structure of (I), with displacement ellipsoids for non-H atoms shown at 50% probability.

**Fig. 2.**
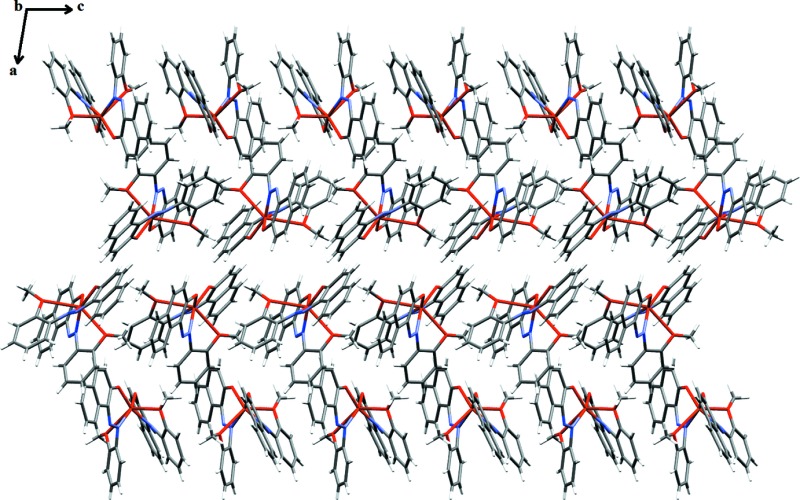
Partial view along the b axis of the crystal packing of the title compound.

. DOI: 10.1107/S2056989015019817/cq2017fig1.tif
The mol­ecular structure of (I), with displacement ellipsoids for non-H atoms shown at 50% probability.

b . DOI: 10.1107/S2056989015019817/cq2017fig2.tif
Partial view along the *b* axis of the crystal packing of the title compound.

CCDC reference: 1432227

Additional supporting information:  crystallographic information; 3D view; checkCIF report

## Figures and Tables

**Table 1:** Hydrogen-bond geometry (, )

*D*H*A*	*D*H	H*A*	*D* *A*	*D*H*A*
C3*B*H3*B*O4*A*	0.93	2.48	3.270(5)	143

## supplementary crystallographic information

### S1. Comment

Azo compounds are highly colored and commonly utilized in textile industries (Millington *et al.*, 2007; Hallas & Choi, 1999), optical data storage (Ho *et al.*,1995) and as sensitizers in dye-sensitized solar cells (DSSCs)(Sharma *et al.*,2008). In previous work, the chelating ligand, (*E*)-1-(*o*-tolyldiazenyl)naphthalen-2-ol, has been used to form complexes on reaction with Cu(OAc)_2_·H_2_O (Tai *et al.*, 2010) and Pd(OAc)_2_ (Lin *et al.*, 2010). Herein, we report the synthesis and crystal structure of a novel copper(II) complex formed on reaction of a similar azoic ligand, (*E*)-1-((2-methoxyphenyl)diazenyl)naphthalen-2-ol (C_17_H_14_N_2_O_2_), with Cu(OAc)_2_·H_2_O.

The molecular structure of the title compound is shown in Figure 1. There are two independent molecules (A and B) in the asymmetric unit, each consisting of a Cu^II^ atom coordinated by two N atoms and four O atoms from two tridentate (*E*)-1-((2-methoxyphenyl)diazenyl)naphthalen-2-ol ligands. The octahedral geometry formed around the Cu^II^ atom (Table 1) is distorted from the ideal due to the Jahn-Teller effect.

Within each molecule, there are C—H···N interactions (Table 1). In the crystal, the two independent molecules are linked *via* weak C—H····O hydrogen bonds (Table 1). These pairs of molecules then stack to form bi-dimensional molecular chains running parallel to [010], as shown in Figure 2.

### S2. Experimental

The title compound (I) was synthesized by the following procedure: (*E*)-1-((2-methoxyphenyl)diazenyl)naphthalen-2-ol (0.55 g, 2.0 mmol) and Cu(O*Ac*)_2_·H_2_O (0.20 g, 1.0 mmol) were stirred at 298 K in methanol (10 ml) for 48 h. Volatile materials were removed under vacuum and the residue was washed twice with CH_2_Cl_2_/hexane (1:5) solution to give a dark solid. Black crystals of the title compound were formed on recrystallization in CH_2_Cl_2_.

### S3. Refinement

The H atoms were placed in idealized positions and constrained to ride on their parent atoms,with C–H = 0.93 Å with *U*_iso_(H) = 1.2Ueq(C) for aromatic hydrogen; C–H = 0.96 Å with *U*_iso_(H) = 1.5Ueq(C) for CH_3_-group. The SQUEEZE alogorithm was used (as implemented in *PLATON*) (Spek, 2015) to correct for the residual electron density between the metal complexes corresponding to disordered methanol.

### Crystal data

**Table:**

[Cu(C_17_H_13_N_2_O_2_)_2_]	*F*(000) = 2552
*M**_r_* = 618.13	*D*_x_ = 1.438 Mg m^−^^3^
Monoclinic, *P*2_1_/*c*	Mo *K*α radiation, λ = 0.71073 Å
Hall symbol: -P 2ybc	Cell parameters from 2052 reflections
*a* = 29.749 (2) Å	θ = 3.1–28.6°
*b* = 12.5171 (9) Å	µ = 0.81 mm^−^^1^
*c* = 15.3565 (12) Å	*T* = 100 K
β = 92.969 (5)°	Block, black
*V* = 5710.7 (8) Å^3^	0.02 × 0.01 × 0.01 mm
*Z* = 8	

### Data collection

**Table:**

Bruker APEXII diffractometer	9009 reflections with *I* > 2σ(*I*)
Radiation source: fine-focus sealed tube	*R*_int_ = 0.122
Graphite monochromator	θ_max_ = 32.3°, θ_min_ = 2.2°
CCD rotation images, thin slices ω scans	*h* = −44→44
76488 measured reflections	*k* = −16→18
20125 independent reflections	*l* = −21→23

### Refinement

**Table:**

Refinement on *F*^2^	Primary atom site location: structure-invariant direct methods
Least-squares matrix: full	Secondary atom site location: difference Fourier map
*R*[*F*^2^ > 2σ(*F*^2^)] = 0.074	Hydrogen site location: inferred from neighbouring sites
*wR*(*F*^2^) = 0.168	H-atom parameters constrained
*S* = 1.01	*w* = 1/[σ^2^(*F*_o_^2^) + (0.0532*P*)^2^ + 4.1986*P*] where *P* = (*F*_o_^2^ + 2*F*_c_^2^)/3
20125 reflections	(Δ/σ)_max_ < 0.001
775 parameters	Δρ_max_ = 0.55 e Å^−^^3^
0 restraints	Δρ_min_ = −1.01 e Å^−^^3^

### Special details

**Table:**

Geometry. Bond distances, angles *etc*. have been calculated using the rounded fractional coordinates. All su's are estimated from the variances of the (full) variance-covariance matrix. The cell e.s.d.'s are taken into account in the estimation of distances, angles and torsion angles
Refinement. Refinement of *F*^2^ against ALL reflections. The weighted *R*-factor *wR* and goodness of fit *S* are based on *F*^2^, conventional *R*-factors *R* are based on *F*, with *F* set to zero for negative *F*^2^. The threshold expression of *F*^2^ > σ(*F*^2^) is used only for calculating *R*-factors(gt) *etc*. and is not relevant to the choice of reflections for refinement. *R*-factors based on *F*^2^ are statistically about twice as large as those based on *F*, and *R*- factors based on ALL data will be even larger.

### Fractional atomic coordinates and isotropic or equivalent isotropic displacement parameters (Å^2^)

**Table:**

	*x*	*y*	*z*	*U*_iso_*/*U*_eq_	
Cu1A	0.16768 (2)	0.07675 (3)	0.64956 (3)	0.0232 (1)	
Cu1B	0.39838 (2)	−0.19075 (3)	0.89627 (3)	0.0326 (2)	
O1A	0.16618 (9)	0.21278 (19)	0.52348 (17)	0.0337 (9)	
O2A	0.20122 (8)	−0.05309 (18)	0.63672 (17)	0.0299 (8)	
O3A	0.10965 (8)	−0.02672 (19)	0.75606 (17)	0.0317 (8)	
O4A	0.21587 (8)	0.14849 (18)	0.71091 (16)	0.0259 (7)	
N1A	0.12605 (9)	0.0277 (2)	0.55555 (18)	0.0230 (9)	
N2A	0.11374 (9)	−0.0690 (2)	0.53966 (18)	0.0245 (9)	
N3A	0.12467 (9)	0.1701 (2)	0.70536 (18)	0.0199 (8)	
N4A	0.13093 (9)	0.2621 (2)	0.73992 (18)	0.0209 (8)	
C1A	0.10128 (12)	0.1064 (3)	0.5062 (2)	0.0241 (11)	
O1B	0.33502 (10)	−0.1213 (2)	0.77943 (17)	0.0401 (10)	
C2A	0.12232 (12)	0.2030 (3)	0.4912 (2)	0.0273 (11)	
O2B	0.42588 (9)	−0.32864 (19)	0.88982 (18)	0.0375 (9)	
C3A	0.09907 (14)	0.2836 (3)	0.4443 (2)	0.0335 (12)	
O3B	0.41311 (12)	−0.1922 (2)	1.06631 (19)	0.0529 (11)	
C4A	0.05525 (14)	0.2654 (3)	0.4142 (2)	0.0377 (14)	
O4B	0.44442 (10)	−0.12394 (19)	0.83389 (18)	0.0398 (9)	
C5A	0.03422 (13)	0.1695 (3)	0.4281 (2)	0.0358 (12)	
C6A	0.05760 (12)	0.0891 (3)	0.4741 (2)	0.0296 (12)	
C7A	0.13682 (12)	−0.1512 (3)	0.5794 (2)	0.0245 (11)	
C8A	0.17995 (13)	−0.1418 (3)	0.6235 (2)	0.0281 (11)	
C9A	0.20128 (14)	−0.2385 (3)	0.6544 (3)	0.0352 (12)	
C10A	0.18092 (15)	−0.3349 (3)	0.6458 (3)	0.0388 (14)	
C11A	0.13786 (14)	−0.3473 (3)	0.6018 (2)	0.0323 (11)	
C12A	0.11583 (13)	−0.2555 (3)	0.5668 (2)	0.0276 (11)	
C13A	0.07372 (13)	−0.2685 (3)	0.5231 (2)	0.0322 (12)	
C14A	0.05408 (15)	−0.3673 (3)	0.5138 (3)	0.0414 (16)	
C15A	0.07519 (15)	−0.4576 (3)	0.5488 (3)	0.0437 (16)	
C16A	0.11664 (15)	−0.4483 (3)	0.5921 (3)	0.0386 (14)	
C17A	0.19126 (13)	0.3024 (3)	0.4981 (3)	0.0413 (14)	
C18A	0.07892 (11)	0.1361 (3)	0.7077 (2)	0.0214 (10)	
C19A	0.07148 (12)	0.0310 (3)	0.7350 (2)	0.0272 (11)	
C20A	0.02787 (13)	−0.0053 (3)	0.7408 (3)	0.0375 (14)	
C21A	−0.00766 (13)	0.0621 (3)	0.7191 (3)	0.0435 (15)	
C22A	−0.00064 (13)	0.1652 (3)	0.6906 (3)	0.0385 (14)	
C23A	0.04326 (12)	0.2023 (3)	0.6857 (2)	0.0281 (11)	
C24A	0.17326 (11)	0.3020 (3)	0.7538 (2)	0.0200 (10)	
C25A	0.21382 (12)	0.2433 (3)	0.7440 (2)	0.0239 (10)	
C26A	0.25519 (11)	0.2914 (3)	0.7758 (2)	0.0263 (11)	
C27A	0.25627 (12)	0.3900 (3)	0.8121 (2)	0.0287 (11)	
C28A	0.21688 (11)	0.4523 (3)	0.8219 (2)	0.0241 (10)	
C29A	0.17510 (11)	0.4079 (3)	0.7938 (2)	0.0206 (10)	
C30A	0.13577 (12)	0.4678 (3)	0.8058 (2)	0.0258 (11)	
C31A	0.13853 (13)	0.5680 (3)	0.8430 (2)	0.0298 (11)	
C32A	0.18007 (13)	0.6118 (3)	0.8693 (2)	0.0308 (11)	
C33A	0.21841 (13)	0.5555 (3)	0.8589 (2)	0.0302 (11)	
C34A	0.10465 (15)	−0.1339 (3)	0.7874 (3)	0.0422 (16)	
N1B	0.33990 (10)	−0.2579 (2)	0.91158 (18)	0.0262 (9)	
N2B	0.33177 (10)	−0.3523 (2)	0.93963 (18)	0.0244 (9)	
N3B	0.38421 (11)	−0.0532 (2)	0.94794 (19)	0.0312 (10)	
N4B	0.38770 (10)	0.0405 (2)	0.91424 (19)	0.0290 (10)	
C1B	0.30027 (13)	−0.1936 (3)	0.8996 (2)	0.0271 (11)	
C2B	0.29852 (14)	−0.1203 (3)	0.8302 (2)	0.0320 (13)	
C3B	0.26207 (16)	−0.0518 (3)	0.8190 (3)	0.0426 (14)	
C4B	0.22736 (15)	−0.0582 (3)	0.8740 (3)	0.0456 (16)	
C5B	0.22840 (15)	−0.1309 (3)	0.9410 (3)	0.0427 (16)	
C6B	0.26509 (13)	−0.1983 (3)	0.9542 (3)	0.0321 (11)	
C7B	0.36599 (12)	−0.4239 (3)	0.9547 (2)	0.0237 (10)	
C8B	0.41092 (13)	−0.4115 (3)	0.9292 (2)	0.0294 (11)	
C9B	0.44149 (13)	−0.4989 (3)	0.9475 (3)	0.0366 (14)	
C10B	0.42835 (14)	−0.5886 (3)	0.9872 (3)	0.0372 (12)	
C11B	0.38393 (13)	−0.6047 (3)	1.0121 (2)	0.0296 (11)	
C12B	0.35182 (12)	−0.5231 (3)	0.9956 (2)	0.0250 (10)	
C13B	0.30741 (14)	−0.5394 (3)	1.0198 (2)	0.0312 (11)	
C14B	0.29580 (15)	−0.6325 (3)	1.0607 (2)	0.0379 (14)	
C15B	0.32742 (17)	−0.7124 (3)	1.0774 (3)	0.0440 (14)	
C16B	0.37014 (16)	−0.6997 (3)	1.0526 (3)	0.0418 (14)	
C17B	0.33199 (18)	−0.0546 (4)	0.7037 (3)	0.0554 (18)	
C18B	0.36165 (14)	−0.0562 (3)	1.0280 (2)	0.0328 (13)	
C19B	0.37675 (17)	−0.1327 (3)	1.0892 (3)	0.0431 (14)	
C20B	0.3548 (2)	−0.1444 (4)	1.1660 (3)	0.0578 (19)	
C21B	0.3191 (2)	−0.0810 (4)	1.1818 (3)	0.064 (2)	
C22B	0.30399 (18)	−0.0044 (3)	1.1220 (3)	0.0541 (18)	
C23B	0.32578 (15)	0.0080 (3)	1.0446 (3)	0.0408 (14)	
C24B	0.41257 (12)	0.0529 (3)	0.8422 (2)	0.0269 (11)	
C25B	0.44239 (13)	−0.0247 (3)	0.8100 (2)	0.0311 (11)	
C26B	0.47397 (13)	0.0098 (3)	0.7488 (3)	0.0357 (11)	
C27B	0.47482 (13)	0.1117 (3)	0.7195 (2)	0.0323 (12)	
C28B	0.44358 (12)	0.1905 (3)	0.7450 (2)	0.0251 (10)	
C29B	0.41151 (12)	0.1612 (3)	0.8060 (2)	0.0235 (10)	
C30B	0.38008 (12)	0.2379 (3)	0.8295 (2)	0.0280 (11)	
C31B	0.38085 (13)	0.3392 (3)	0.7941 (3)	0.0317 (11)	
C32B	0.41280 (13)	0.3679 (3)	0.7359 (2)	0.0301 (11)	
C33B	0.44360 (13)	0.2955 (3)	0.7117 (2)	0.0315 (12)	
C34B	0.4358 (2)	−0.2576 (5)	1.1305 (4)	0.079 (2)	
H3A	0.11300	0.34840	0.43360	0.0400*	
H4A	0.03960	0.31920	0.38390	0.0450*	
H5A	0.00470	0.15850	0.40710	0.0430*	
H6A	0.04380	0.02370	0.48320	0.0350*	
H9A	0.23000	−0.23480	0.68100	0.0420*	
H10A	0.19540	−0.39490	0.66940	0.0470*	
H13A	0.05870	−0.20890	0.49990	0.0390*	
H14A	0.02630	−0.37390	0.48370	0.0500*	
H15A	0.06140	−0.52410	0.54310	0.0530*	
H16A	0.13090	−0.50890	0.61530	0.0460*	
H17A	0.22100	0.29900	0.52520	0.0620*	
H17B	0.19310	0.30240	0.43580	0.0620*	
H17C	0.17670	0.36660	0.51590	0.0620*	
H20A	0.02250	−0.07470	0.75920	0.0450*	
H21A	−0.03700	0.03760	0.72370	0.0520*	
H22A	−0.02490	0.20920	0.67500	0.0460*	
H23A	0.04850	0.27190	0.66760	0.0340*	
H26A	0.28200	0.25400	0.77130	0.0320*	
H27A	0.28390	0.41810	0.83150	0.0350*	
H30A	0.10780	0.43960	0.78850	0.0310*	
H31A	0.11240	0.60670	0.85060	0.0360*	
H32A	0.18150	0.67960	0.89410	0.0370*	
H33A	0.24610	0.58530	0.87640	0.0360*	
H34A	0.13380	−0.16480	0.79960	0.0640*	
H34B	0.08830	−0.13280	0.83970	0.0640*	
H34C	0.08840	−0.17570	0.74380	0.0640*	
H3B	0.26120	−0.00170	0.77430	0.0510*	
H4B	0.20280	−0.01280	0.86590	0.0550*	
H5B	0.20450	−0.13490	0.97750	0.0520*	
H6B	0.26590	−0.24680	1.00010	0.0380*	
H9B	0.47110	−0.49310	0.93150	0.0440*	
H10B	0.44950	−0.64220	0.99880	0.0440*	
H13B	0.28580	−0.48700	1.00820	0.0370*	
H14B	0.26640	−0.64200	1.07730	0.0450*	
H15B	0.31930	−0.77460	1.10570	0.0530*	
H16B	0.39080	−0.75470	1.06250	0.0500*	
H17D	0.35910	−0.06100	0.67280	0.0830*	
H17E	0.30670	−0.07630	0.66650	0.0830*	
H17F	0.32810	0.01840	0.72110	0.0830*	
H20B	0.36450	−0.19550	1.20670	0.0700*	
H21B	0.30450	−0.08910	1.23350	0.0760*	
H22B	0.27950	0.03840	1.13350	0.0650*	
H23B	0.31600	0.05960	1.00430	0.0490*	
H26B	0.49450	−0.03910	0.72860	0.0430*	
H27B	0.49670	0.13110	0.68130	0.0390*	
H30B	0.35860	0.22050	0.86910	0.0340*	
H31B	0.35950	0.38900	0.80960	0.0380*	
H32B	0.41310	0.43690	0.71350	0.0360*	
H33B	0.46500	0.31520	0.67260	0.0380*	
H34D	0.46030	−0.29410	1.10510	0.1180*	
H34E	0.44720	−0.21380	1.17800	0.1180*	
H34F	0.41510	−0.30900	1.15190	0.1180*	

### Atomic displacement parameters (Å^2^)

**Table:**

	*U*^11^	*U*^22^	*U*^33^	*U*^12^	*U*^13^	*U*^23^
Cu1A	0.0208 (2)	0.0217 (2)	0.0270 (2)	0.0036 (2)	0.0015 (2)	−0.0017 (2)
Cu1B	0.0482 (3)	0.0193 (2)	0.0317 (3)	−0.0050 (2)	0.0153 (2)	0.0016 (2)
O1A	0.0329 (15)	0.0269 (14)	0.0412 (16)	−0.0035 (11)	0.0016 (13)	0.0066 (12)
O2A	0.0243 (13)	0.0241 (13)	0.0410 (16)	0.0042 (11)	−0.0008 (12)	−0.0036 (11)
O3A	0.0276 (14)	0.0257 (13)	0.0420 (16)	−0.0005 (11)	0.0050 (12)	0.0062 (12)
O4A	0.0198 (12)	0.0252 (12)	0.0323 (14)	0.0055 (10)	−0.0014 (11)	−0.0037 (11)
N1A	0.0249 (15)	0.0220 (15)	0.0221 (15)	0.0021 (12)	0.0028 (12)	0.0010 (12)
N2A	0.0259 (15)	0.0237 (15)	0.0242 (15)	0.0022 (13)	0.0040 (12)	−0.0052 (12)
N3A	0.0174 (14)	0.0203 (14)	0.0222 (15)	−0.0009 (11)	0.0027 (12)	0.0004 (11)
N4A	0.0186 (14)	0.0209 (14)	0.0236 (15)	−0.0004 (12)	0.0053 (12)	0.0023 (12)
C1A	0.0278 (19)	0.0244 (18)	0.0205 (18)	0.0045 (15)	0.0057 (15)	0.0004 (14)
O1B	0.062 (2)	0.0337 (15)	0.0253 (14)	0.0052 (14)	0.0095 (14)	0.0089 (12)
C2A	0.030 (2)	0.0293 (19)	0.0229 (19)	0.0050 (16)	0.0051 (16)	−0.0021 (15)
O2B	0.0415 (16)	0.0250 (14)	0.0476 (17)	−0.0048 (12)	0.0188 (14)	0.0020 (12)
C3A	0.047 (2)	0.028 (2)	0.026 (2)	0.0077 (18)	0.0069 (18)	0.0006 (16)
O3B	0.081 (2)	0.0451 (18)	0.0324 (17)	0.0039 (17)	0.0005 (16)	0.0106 (14)
C4A	0.047 (3)	0.040 (2)	0.026 (2)	0.018 (2)	0.0022 (19)	0.0026 (17)
O4B	0.0539 (18)	0.0217 (13)	0.0459 (17)	0.0025 (13)	0.0224 (14)	0.0108 (12)
C5A	0.032 (2)	0.046 (2)	0.029 (2)	0.0153 (19)	−0.0009 (17)	−0.0020 (18)
C6A	0.030 (2)	0.031 (2)	0.028 (2)	0.0048 (16)	0.0026 (16)	−0.0051 (16)
C7A	0.031 (2)	0.0194 (17)	0.0234 (18)	0.0014 (15)	0.0051 (15)	−0.0032 (14)
C8A	0.036 (2)	0.0242 (19)	0.0243 (19)	0.0077 (16)	0.0049 (16)	−0.0071 (15)
C9A	0.039 (2)	0.027 (2)	0.039 (2)	0.0142 (18)	−0.0026 (18)	−0.0011 (17)
C10A	0.056 (3)	0.026 (2)	0.034 (2)	0.0132 (19)	−0.002 (2)	−0.0044 (17)
C11A	0.043 (2)	0.0247 (19)	0.030 (2)	0.0051 (17)	0.0086 (18)	−0.0039 (16)
C12A	0.038 (2)	0.0255 (19)	0.0200 (18)	0.0023 (16)	0.0089 (16)	−0.0042 (14)
C13A	0.032 (2)	0.032 (2)	0.033 (2)	0.0043 (17)	0.0064 (18)	−0.0031 (17)
C14A	0.043 (3)	0.036 (2)	0.046 (3)	−0.003 (2)	0.009 (2)	−0.009 (2)
C15A	0.054 (3)	0.029 (2)	0.049 (3)	−0.012 (2)	0.011 (2)	−0.0098 (19)
C16A	0.057 (3)	0.0210 (19)	0.039 (2)	0.0036 (18)	0.014 (2)	−0.0021 (17)
C17A	0.033 (2)	0.034 (2)	0.057 (3)	−0.0068 (18)	0.004 (2)	0.009 (2)
C18A	0.0191 (17)	0.0231 (17)	0.0223 (17)	−0.0023 (14)	0.0046 (14)	−0.0065 (14)
C19A	0.0253 (19)	0.0302 (19)	0.0265 (19)	−0.0021 (16)	0.0043 (16)	−0.0079 (15)
C20A	0.033 (2)	0.033 (2)	0.047 (3)	−0.0105 (18)	0.0069 (19)	−0.0050 (19)
C21A	0.0205 (19)	0.049 (3)	0.061 (3)	−0.0148 (19)	0.0031 (19)	−0.016 (2)
C22A	0.022 (2)	0.044 (2)	0.049 (3)	0.0046 (18)	−0.0024 (18)	−0.013 (2)
C23A	0.0256 (19)	0.0271 (19)	0.031 (2)	0.0011 (16)	−0.0035 (16)	−0.0065 (16)
C24A	0.0196 (17)	0.0198 (16)	0.0207 (17)	−0.0013 (14)	0.0010 (14)	0.0018 (13)
C25A	0.0253 (18)	0.0264 (18)	0.0202 (18)	0.0013 (15)	0.0028 (15)	0.0030 (14)
C26A	0.0171 (17)	0.032 (2)	0.030 (2)	0.0020 (15)	0.0024 (15)	−0.0008 (16)
C27A	0.0215 (18)	0.033 (2)	0.032 (2)	−0.0058 (15)	0.0047 (16)	−0.0006 (16)
C28A	0.0243 (18)	0.0280 (18)	0.0204 (17)	−0.0064 (15)	0.0052 (14)	0.0011 (14)
C29A	0.0250 (17)	0.0215 (17)	0.0154 (16)	−0.0040 (14)	0.0032 (14)	0.0015 (13)
C30A	0.0234 (18)	0.0249 (18)	0.029 (2)	−0.0061 (15)	0.0013 (15)	−0.0014 (15)
C31A	0.032 (2)	0.0270 (19)	0.031 (2)	0.0040 (17)	0.0083 (16)	−0.0007 (16)
C32A	0.035 (2)	0.0274 (19)	0.030 (2)	−0.0083 (17)	0.0018 (17)	−0.0073 (16)
C33A	0.032 (2)	0.032 (2)	0.0266 (19)	−0.0077 (17)	0.0028 (16)	−0.0035 (16)
C34A	0.053 (3)	0.030 (2)	0.045 (3)	0.001 (2)	0.016 (2)	0.0089 (19)
N1B	0.0393 (18)	0.0193 (14)	0.0204 (15)	−0.0003 (13)	0.0059 (13)	0.0026 (12)
N2B	0.0352 (17)	0.0185 (14)	0.0195 (15)	−0.0012 (13)	0.0004 (13)	0.0033 (12)
N3B	0.046 (2)	0.0238 (16)	0.0246 (16)	−0.0053 (14)	0.0101 (15)	0.0012 (13)
N4B	0.0372 (18)	0.0228 (15)	0.0277 (17)	−0.0073 (13)	0.0093 (14)	0.0008 (13)
C1B	0.034 (2)	0.0208 (17)	0.0264 (19)	0.0017 (16)	−0.0001 (16)	−0.0059 (15)
C2B	0.050 (3)	0.0202 (18)	0.025 (2)	0.0039 (17)	−0.0042 (18)	0.0014 (15)
C3B	0.067 (3)	0.026 (2)	0.033 (2)	0.002 (2)	−0.016 (2)	−0.0009 (17)
C4B	0.046 (3)	0.033 (2)	0.056 (3)	0.015 (2)	−0.014 (2)	−0.007 (2)
C5B	0.041 (3)	0.038 (2)	0.049 (3)	0.003 (2)	0.001 (2)	−0.013 (2)
C6B	0.037 (2)	0.0258 (19)	0.033 (2)	−0.0012 (17)	−0.0021 (18)	−0.0051 (16)
C7B	0.0325 (19)	0.0178 (16)	0.0205 (17)	−0.0008 (15)	0.0000 (15)	0.0008 (14)
C8B	0.038 (2)	0.0217 (18)	0.029 (2)	−0.0043 (16)	0.0055 (17)	−0.0013 (15)
C9B	0.031 (2)	0.033 (2)	0.046 (3)	0.0019 (18)	0.0036 (19)	0.0016 (18)
C10B	0.042 (2)	0.030 (2)	0.039 (2)	0.0066 (18)	−0.0032 (19)	0.0049 (18)
C11B	0.044 (2)	0.0201 (18)	0.0240 (19)	−0.0012 (16)	−0.0051 (17)	0.0027 (14)
C12B	0.038 (2)	0.0179 (17)	0.0190 (17)	−0.0040 (15)	0.0020 (16)	−0.0022 (14)
C13B	0.045 (2)	0.0281 (19)	0.0208 (18)	−0.0070 (17)	0.0040 (17)	−0.0022 (15)
C14B	0.056 (3)	0.030 (2)	0.029 (2)	−0.015 (2)	0.0139 (19)	−0.0025 (17)
C15B	0.076 (3)	0.023 (2)	0.034 (2)	−0.013 (2)	0.012 (2)	−0.0007 (17)
C16B	0.069 (3)	0.023 (2)	0.033 (2)	0.003 (2)	−0.001 (2)	0.0083 (17)
C17B	0.077 (4)	0.057 (3)	0.032 (2)	−0.009 (3)	0.000 (2)	0.018 (2)
C18B	0.053 (3)	0.0209 (18)	0.0258 (19)	−0.0163 (18)	0.0133 (18)	−0.0050 (15)
C19B	0.076 (3)	0.028 (2)	0.026 (2)	−0.017 (2)	0.009 (2)	−0.0039 (17)
C20B	0.118 (5)	0.034 (2)	0.023 (2)	−0.025 (3)	0.020 (3)	−0.0029 (19)
C21B	0.115 (5)	0.039 (3)	0.040 (3)	−0.039 (3)	0.039 (3)	−0.014 (2)
C22B	0.080 (4)	0.037 (2)	0.049 (3)	−0.021 (2)	0.038 (3)	−0.015 (2)
C23B	0.068 (3)	0.0243 (19)	0.032 (2)	−0.013 (2)	0.020 (2)	−0.0066 (17)
C24B	0.034 (2)	0.0242 (18)	0.0229 (18)	−0.0057 (15)	0.0054 (16)	0.0031 (14)
C25B	0.037 (2)	0.0262 (19)	0.031 (2)	−0.0033 (17)	0.0103 (17)	0.0035 (16)
C26B	0.039 (2)	0.0267 (19)	0.043 (2)	0.0047 (17)	0.0182 (19)	0.0041 (17)
C27B	0.032 (2)	0.032 (2)	0.034 (2)	−0.0023 (17)	0.0118 (17)	0.0028 (17)
C28B	0.0255 (18)	0.0227 (17)	0.0274 (19)	−0.0033 (15)	0.0045 (15)	0.0011 (15)
C29B	0.0251 (18)	0.0215 (17)	0.0238 (18)	−0.0045 (14)	0.0016 (15)	−0.0001 (14)
C30B	0.0273 (19)	0.0269 (19)	0.030 (2)	−0.0033 (16)	0.0047 (16)	0.0015 (16)
C31B	0.029 (2)	0.0271 (19)	0.039 (2)	0.0022 (16)	0.0009 (18)	0.0012 (16)
C32B	0.037 (2)	0.0210 (18)	0.032 (2)	−0.0026 (16)	−0.0014 (17)	0.0052 (16)
C33B	0.030 (2)	0.031 (2)	0.034 (2)	−0.0062 (17)	0.0064 (17)	0.0084 (17)
C34B	0.088 (4)	0.083 (4)	0.062 (4)	0.006 (3)	−0.019 (3)	0.036 (3)

### Geometric parameters (Å, º)

**Table:**

Cu1A—O1A	2.577 (3)	C15A—H15A	0.9300
Cu1A—O2A	1.923 (2)	C16A—H16A	0.9300
Cu1A—O3A	2.761 (3)	C17A—H17C	0.9600
Cu1A—O4A	1.900 (2)	C17A—H17B	0.9600
Cu1A—N1A	1.952 (3)	C17A—H17A	0.9600
Cu1A—N3A	1.962 (3)	C20A—H20A	0.9300
Cu1B—N3B	1.951 (3)	C21A—H21A	0.9300
Cu1B—O3B	2.625 (3)	C22A—H22A	0.9300
Cu1B—O1B	2.679 (3)	C23A—H23A	0.9300
Cu1B—O2B	1.915 (2)	C26A—H26A	0.9300
Cu1B—O4B	1.905 (3)	C27A—H27A	0.9300
Cu1B—N1B	1.957 (3)	C30A—H30A	0.9300
O1A—C17A	1.413 (5)	C31A—H31A	0.9300
O1A—C2A	1.377 (4)	C32A—H32A	0.9300
O2A—C8A	1.289 (4)	C33A—H33A	0.9300
O3A—C34A	1.436 (5)	C34A—H34A	0.9600
O3A—C19A	1.370 (4)	C34A—H34B	0.9600
O4A—C25A	1.294 (4)	C34A—H34C	0.9600
N1A—C1A	1.425 (4)	C1B—C2B	1.405 (5)
N1A—N2A	1.284 (4)	C1B—C6B	1.376 (5)
N2A—C7A	1.364 (4)	C2B—C3B	1.386 (6)
N3A—N4A	1.278 (4)	C3B—C4B	1.370 (7)
N3A—C18A	1.428 (4)	C4B—C5B	1.373 (6)
N4A—C24A	1.361 (4)	C5B—C6B	1.386 (6)
C1A—C6A	1.383 (5)	C7B—C8B	1.421 (5)
C1A—C2A	1.386 (5)	C7B—C12B	1.463 (5)
O1B—C2B	1.369 (5)	C8B—C9B	1.441 (5)
O1B—C17B	1.431 (5)	C9B—C10B	1.345 (6)
C2A—C3A	1.401 (5)	C10B—C11B	1.409 (6)
O2B—C8B	1.291 (4)	C11B—C12B	1.412 (5)
C3A—C4A	1.379 (6)	C11B—C16B	1.413 (5)
O3B—C19B	1.374 (6)	C12B—C13B	1.406 (5)
O3B—C34B	1.424 (7)	C13B—C14B	1.376 (5)
C4A—C5A	1.375 (5)	C14B—C15B	1.388 (6)
O4B—C25B	1.296 (4)	C15B—C16B	1.355 (7)
C5A—C6A	1.395 (5)	C18B—C19B	1.399 (6)
C7A—C8A	1.424 (5)	C18B—C23B	1.370 (6)
C7A—C12A	1.456 (5)	C19B—C20B	1.385 (7)
C8A—C9A	1.436 (5)	C20B—C21B	1.358 (8)
C9A—C10A	1.354 (5)	C21B—C22B	1.386 (7)
C10A—C11A	1.426 (6)	C22B—C23B	1.392 (7)
C11A—C16A	1.418 (5)	C24B—C25B	1.421 (5)
C11A—C12A	1.415 (5)	C24B—C29B	1.465 (5)
C12A—C13A	1.400 (5)	C25B—C26B	1.430 (5)
C13A—C14A	1.372 (5)	C26B—C27B	1.353 (5)
C14A—C15A	1.388 (6)	C27B—C28B	1.424 (5)
C15A—C16A	1.375 (6)	C28B—C29B	1.419 (5)
C18A—C23A	1.374 (5)	C28B—C33B	1.410 (5)
C18A—C19A	1.402 (5)	C29B—C30B	1.400 (5)
C19A—C20A	1.382 (5)	C30B—C31B	1.380 (5)
C20A—C21A	1.380 (5)	C31B—C32B	1.385 (5)
C21A—C22A	1.382 (5)	C32B—C33B	1.354 (5)
C22A—C23A	1.392 (5)	C3B—H3B	0.9300
C24A—C25A	1.427 (5)	C4B—H4B	0.9300
C24A—C29A	1.461 (5)	C5B—H5B	0.9300
C25A—C26A	1.433 (5)	C6B—H6B	0.9300
C26A—C27A	1.354 (5)	C9B—H9B	0.9300
C27A—C28A	1.422 (5)	C10B—H10B	0.9300
C28A—C29A	1.409 (5)	C13B—H13B	0.9300
C28A—C33A	1.411 (5)	C14B—H14B	0.9300
C29A—C30A	1.410 (5)	C15B—H15B	0.9300
C30A—C31A	1.379 (5)	C16B—H16B	0.9300
C31A—C32A	1.393 (5)	C17B—H17D	0.9600
C32A—C33A	1.357 (5)	C17B—H17E	0.9600
N1B—C1B	1.432 (5)	C17B—H17F	0.9600
N1B—N2B	1.285 (4)	C20B—H20B	0.9300
N2B—C7B	1.367 (5)	C21B—H21B	0.9300
C3A—H3A	0.9300	C22B—H22B	0.9300
N3B—N4B	1.288 (4)	C23B—H23B	0.9300
N3B—C18B	1.431 (5)	C26B—H26B	0.9300
C4A—H4A	0.9300	C27B—H27B	0.9300
N4B—C24B	1.371 (4)	C30B—H30B	0.9300
C5A—H5A	0.9300	C31B—H31B	0.9300
C6A—H6A	0.9300	C32B—H32B	0.9300
C9A—H9A	0.9300	C33B—H33B	0.9300
C10A—H10A	0.9300	C34B—H34D	0.9600
C13A—H13A	0.9300	C34B—H34E	0.9600
C14A—H14A	0.9300	C34B—H34F	0.9600
			
O1A—Cu1A—O2A	118.06 (10)	O1A—C17A—H17B	109.00
O1A—Cu1A—O3A	140.16 (8)	O1A—C17A—H17C	109.00
O1A—Cu1A—O4A	92.50 (9)	H17A—C17A—H17B	109.00
O1A—Cu1A—N1A	70.52 (10)	H17A—C17A—H17C	109.00
O1A—Cu1A—N3A	87.04 (10)	H17B—C17A—H17C	109.00
O2A—Cu1A—O3A	90.46 (9)	C19A—C20A—H20A	120.00
O2A—Cu1A—O4A	93.91 (10)	C21A—C20A—H20A	120.00
O2A—Cu1A—N1A	88.34 (11)	C20A—C21A—H21A	119.00
O2A—Cu1A—N3A	154.40 (11)	C22A—C21A—H21A	119.00
O3A—Cu1A—O4A	114.01 (9)	C21A—C22A—H22A	120.00
O3A—Cu1A—N1A	84.17 (10)	C23A—C22A—H22A	120.00
O3A—Cu1A—N3A	65.09 (9)	C18A—C23A—H23A	120.00
O4A—Cu1A—N1A	161.64 (11)	C22A—C23A—H23A	120.00
O4A—Cu1A—N3A	89.68 (11)	C25A—C26A—H26A	119.00
N1A—Cu1A—N3A	96.15 (11)	C27A—C26A—H26A	119.00
O1B—Cu1B—N1B	67.72 (10)	C26A—C27A—H27A	119.00
O1B—Cu1B—N3B	80.02 (11)	C28A—C27A—H27A	119.00
O2B—Cu1B—O3B	89.74 (10)	C29A—C30A—H30A	120.00
O2B—Cu1B—O4B	92.83 (11)	C31A—C30A—H30A	120.00
O2B—Cu1B—N1B	90.23 (11)	C30A—C31A—H31A	120.00
O2B—Cu1B—N3B	156.69 (12)	C32A—C31A—H31A	120.00
O3B—Cu1B—O4B	114.58 (11)	C31A—C32A—H32A	120.00
O3B—Cu1B—N1B	88.92 (11)	C33A—C32A—H32A	120.00
O3B—Cu1B—N3B	68.29 (10)	C28A—C33A—H33A	120.00
O4B—Cu1B—N1B	156.30 (12)	C32A—C33A—H33A	120.00
O4B—Cu1B—N3B	89.61 (12)	O3A—C34A—H34A	110.00
N1B—Cu1B—N3B	96.78 (12)	O3A—C34A—H34B	109.00
O1B—Cu1B—O2B	123.06 (10)	O3A—C34A—H34C	109.00
O1B—Cu1B—O3B	138.05 (10)	H34A—C34A—H34B	110.00
O1B—Cu1B—O4B	91.15 (11)	H34A—C34A—H34C	110.00
Cu1A—O1A—C17A	138.0 (2)	H34B—C34A—H34C	109.00
C2A—O1A—C17A	118.4 (3)	N1B—C1B—C2B	117.6 (3)
Cu1A—O1A—C2A	101.01 (19)	N1B—C1B—C6B	123.0 (3)
Cu1A—O2A—C8A	119.4 (2)	C2B—C1B—C6B	119.4 (4)
Cu1A—O3A—C19A	98.56 (19)	O1B—C2B—C1B	115.3 (3)
Cu1A—O3A—C34A	135.7 (2)	O1B—C2B—C3B	125.0 (3)
C19A—O3A—C34A	118.2 (3)	C1B—C2B—C3B	119.7 (4)
Cu1A—O4A—C25A	125.4 (2)	C2B—C3B—C4B	119.8 (4)
Cu1A—N1A—C1A	117.8 (2)	C3B—C4B—C5B	120.8 (4)
N2A—N1A—C1A	114.6 (3)	C4B—C5B—C6B	120.0 (4)
Cu1A—N1A—N2A	126.9 (2)	C1B—C6B—C5B	120.2 (4)
N1A—N2A—C7A	119.6 (3)	N2B—C7B—C8B	125.8 (3)
N4A—N3A—C18A	112.1 (3)	N2B—C7B—C12B	113.6 (3)
Cu1A—N3A—C18A	118.5 (2)	C8B—C7B—C12B	120.5 (3)
Cu1A—N3A—N4A	129.3 (2)	O2B—C8B—C7B	124.7 (3)
N3A—N4A—C24A	120.6 (3)	O2B—C8B—C9B	118.2 (3)
N1A—C1A—C2A	117.8 (3)	C7B—C8B—C9B	117.2 (3)
N1A—C1A—C6A	122.1 (3)	C8B—C9B—C10B	121.8 (4)
C2A—C1A—C6A	120.1 (3)	C9B—C10B—C11B	122.8 (4)
C2B—O1B—C17B	116.1 (3)	C10B—C11B—C12B	118.9 (3)
Cu1B—O1B—C2B	100.0 (2)	C10B—C11B—C16B	122.5 (4)
Cu1B—O1B—C17B	138.3 (3)	C12B—C11B—C16B	118.6 (4)
O1A—C2A—C1A	116.4 (3)	C7B—C12B—C11B	118.9 (3)
O1A—C2A—C3A	123.7 (3)	C7B—C12B—C13B	122.2 (3)
C1A—C2A—C3A	119.9 (3)	C11B—C12B—C13B	118.9 (3)
Cu1B—O2B—C8B	122.8 (2)	C12B—C13B—C14B	120.3 (4)
C2A—C3A—C4A	119.1 (3)	C13B—C14B—C15B	120.7 (4)
Cu1B—O3B—C19B	99.1 (2)	C14B—C15B—C16B	120.0 (4)
Cu1B—O3B—C34B	137.8 (3)	C11B—C16B—C15B	121.4 (4)
C19B—O3B—C34B	119.1 (4)	N3B—C18B—C19B	116.5 (4)
C3A—C4A—C5A	121.5 (3)	N3B—C18B—C23B	123.3 (3)
Cu1B—O4B—C25B	122.6 (2)	C19B—C18B—C23B	120.2 (4)
C4A—C5A—C6A	119.3 (3)	O3B—C19B—C18B	115.4 (4)
C1A—C6A—C5A	120.2 (3)	O3B—C19B—C20B	124.9 (4)
N2A—C7A—C8A	124.8 (3)	C18B—C19B—C20B	119.7 (4)
C8A—C7A—C12A	120.5 (3)	C19B—C20B—C21B	119.9 (4)
N2A—C7A—C12A	114.5 (3)	C20B—C21B—C22B	121.0 (5)
O2A—C8A—C7A	124.7 (3)	C21B—C22B—C23B	119.6 (4)
O2A—C8A—C9A	118.0 (3)	C18B—C23B—C22B	119.7 (4)
C7A—C8A—C9A	117.3 (3)	N4B—C24B—C25B	125.2 (3)
C8A—C9A—C10A	122.1 (4)	N4B—C24B—C29B	114.1 (3)
C9A—C10A—C11A	122.0 (4)	C25B—C24B—C29B	120.1 (3)
C12A—C11A—C16A	119.3 (4)	O4B—C25B—C24B	125.3 (3)
C10A—C11A—C12A	118.6 (3)	O4B—C25B—C26B	116.9 (3)
C10A—C11A—C16A	122.1 (4)	C24B—C25B—C26B	117.7 (3)
C7A—C12A—C11A	119.4 (3)	C25B—C26B—C27B	121.8 (4)
C7A—C12A—C13A	122.4 (3)	C26B—C27B—C28B	122.5 (3)
C11A—C12A—C13A	118.2 (3)	C27B—C28B—C29B	118.2 (3)
C12A—C13A—C14A	121.4 (4)	C27B—C28B—C33B	122.2 (3)
C13A—C14A—C15A	120.8 (4)	C29B—C28B—C33B	119.5 (3)
C14A—C15A—C16A	119.7 (4)	C24B—C29B—C28B	119.2 (3)
C11A—C16A—C15A	120.6 (4)	C24B—C29B—C30B	122.6 (3)
N3A—C18A—C19A	116.9 (3)	C28B—C29B—C30B	118.3 (3)
N3A—C18A—C23A	122.6 (3)	C29B—C30B—C31B	120.2 (3)
C19A—C18A—C23A	120.5 (3)	C30B—C31B—C32B	121.2 (4)
O3A—C19A—C18A	115.1 (3)	C31B—C32B—C33B	120.0 (3)
O3A—C19A—C20A	125.5 (3)	C28B—C33B—C32B	120.7 (3)
C18A—C19A—C20A	119.4 (3)	C2B—C3B—H3B	120.00
C19A—C20A—C21A	119.6 (4)	C4B—C3B—H3B	120.00
C20A—C21A—C22A	121.4 (4)	C3B—C4B—H4B	120.00
C21A—C22A—C23A	119.1 (4)	C5B—C4B—H4B	120.00
C18A—C23A—C22A	120.0 (3)	C4B—C5B—H5B	120.00
N4A—C24A—C29A	114.4 (3)	C6B—C5B—H5B	120.00
C25A—C24A—C29A	119.9 (3)	C1B—C6B—H6B	120.00
N4A—C24A—C25A	125.1 (3)	C5B—C6B—H6B	120.00
O4A—C25A—C24A	124.8 (3)	C8B—C9B—H9B	119.00
C24A—C25A—C26A	117.6 (3)	C10B—C9B—H9B	119.00
O4A—C25A—C26A	117.5 (3)	C9B—C10B—H10B	119.00
C25A—C26A—C27A	121.7 (3)	C11B—C10B—H10B	119.00
C26A—C27A—C28A	122.8 (3)	C12B—C13B—H13B	120.00
C27A—C28A—C29A	118.0 (3)	C14B—C13B—H13B	120.00
C27A—C28A—C33A	122.4 (3)	C13B—C14B—H14B	120.00
C29A—C28A—C33A	119.6 (3)	C15B—C14B—H14B	120.00
C28A—C29A—C30A	118.4 (3)	C14B—C15B—H15B	120.00
C24A—C29A—C30A	121.6 (3)	C16B—C15B—H15B	120.00
C24A—C29A—C28A	120.0 (3)	C11B—C16B—H16B	119.00
C29A—C30A—C31A	120.4 (3)	C15B—C16B—H16B	119.00
C30A—C31A—C32A	120.7 (3)	O1B—C17B—H17D	110.00
C31A—C32A—C33A	120.0 (3)	O1B—C17B—H17E	109.00
C28A—C33A—C32A	120.9 (3)	O1B—C17B—H17F	109.00
Cu1B—N1B—N2B	128.3 (2)	H17D—C17B—H17E	110.00
Cu1B—N1B—C1B	118.3 (2)	H17D—C17B—H17F	109.00
N2B—N1B—C1B	113.1 (3)	H17E—C17B—H17F	109.00
N1B—N2B—C7B	120.5 (3)	C19B—C20B—H20B	120.00
C2A—C3A—H3A	120.00	C21B—C20B—H20B	120.00
C4A—C3A—H3A	120.00	C20B—C21B—H21B	120.00
N4B—N3B—C18B	114.8 (3)	C22B—C21B—H21B	119.00
Cu1B—N3B—N4B	128.1 (2)	C21B—C22B—H22B	120.00
Cu1B—N3B—C18B	116.6 (2)	C23B—C22B—H22B	120.00
C5A—C4A—H4A	119.00	C18B—C23B—H23B	120.00
C3A—C4A—H4A	119.00	C22B—C23B—H23B	120.00
N3B—N4B—C24B	119.0 (3)	C25B—C26B—H26B	119.00
C4A—C5A—H5A	120.00	C27B—C26B—H26B	119.00
C6A—C5A—H5A	120.00	C26B—C27B—H27B	119.00
C1A—C6A—H6A	120.00	C28B—C27B—H27B	119.00
C5A—C6A—H6A	120.00	C29B—C30B—H30B	120.00
C10A—C9A—H9A	119.00	C31B—C30B—H30B	120.00
C8A—C9A—H9A	119.00	C30B—C31B—H31B	119.00
C9A—C10A—H10A	119.00	C32B—C31B—H31B	119.00
C11A—C10A—H10A	119.00	C31B—C32B—H32B	120.00
C14A—C13A—H13A	119.00	C33B—C32B—H32B	120.00
C12A—C13A—H13A	119.00	C28B—C33B—H33B	120.00
C13A—C14A—H14A	120.00	C32B—C33B—H33B	120.00
C15A—C14A—H14A	120.00	O3B—C34B—H34D	110.00
C16A—C15A—H15A	120.00	O3B—C34B—H34E	109.00
C14A—C15A—H15A	120.00	O3B—C34B—H34F	109.00
C15A—C16A—H16A	120.00	H34D—C34B—H34E	110.00
C11A—C16A—H16A	120.00	H34D—C34B—H34F	110.00
O1A—C17A—H17A	109.00	H34E—C34B—H34F	109.00
			
O2A—Cu1A—O1A—C2A	109.2 (2)	C4A—C5A—C6A—C1A	0.8 (5)
O2A—Cu1A—O1A—C17A	−90.9 (3)	C12A—C7A—C8A—O2A	179.2 (3)
O3A—Cu1A—O1A—C2A	−21.5 (2)	N2A—C7A—C8A—O2A	5.1 (5)
O3A—Cu1A—O1A—C17A	138.4 (3)	N2A—C7A—C8A—C9A	−174.1 (3)
O4A—Cu1A—O1A—C2A	−155.0 (2)	N2A—C7A—C12A—C13A	−4.7 (5)
O4A—Cu1A—O1A—C17A	4.9 (3)	C12A—C7A—C8A—C9A	−0.1 (5)
N1A—Cu1A—O1A—C2A	32.1 (2)	N2A—C7A—C12A—C11A	177.2 (3)
N1A—Cu1A—O1A—C17A	−168.0 (4)	C8A—C7A—C12A—C11A	2.5 (5)
N3A—Cu1A—O1A—C2A	−65.5 (2)	C8A—C7A—C12A—C13A	−179.4 (3)
N3A—Cu1A—O1A—C17A	94.4 (3)	C7A—C8A—C9A—C10A	−2.7 (6)
O1A—Cu1A—O2A—C8A	−107.5 (2)	O2A—C8A—C9A—C10A	178.0 (4)
O3A—Cu1A—O2A—C8A	43.5 (2)	C8A—C9A—C10A—C11A	3.1 (7)
O4A—Cu1A—O2A—C8A	157.6 (2)	C9A—C10A—C11A—C12A	−0.5 (6)
N1A—Cu1A—O2A—C8A	−40.6 (2)	C9A—C10A—C11A—C16A	179.5 (4)
N3A—Cu1A—O2A—C8A	60.2 (4)	C10A—C11A—C16A—C15A	−179.6 (4)
O1A—Cu1A—O3A—C19A	−11.0 (2)	C10A—C11A—C12A—C7A	−2.3 (5)
O1A—Cu1A—O3A—C34A	136.0 (3)	C10A—C11A—C12A—C13A	179.5 (3)
O2A—Cu1A—O3A—C19A	−149.1 (2)	C16A—C11A—C12A—C7A	177.8 (3)
O2A—Cu1A—O3A—C34A	−2.1 (3)	C16A—C11A—C12A—C13A	−0.4 (5)
O4A—Cu1A—O3A—C19A	116.43 (19)	C12A—C11A—C16A—C15A	0.3 (6)
O4A—Cu1A—O3A—C34A	−96.6 (3)	C11A—C12A—C13A—C14A	−0.2 (5)
N1A—Cu1A—O3A—C19A	−60.8 (2)	C7A—C12A—C13A—C14A	−178.4 (3)
N1A—Cu1A—O3A—C34A	86.2 (3)	C12A—C13A—C14A—C15A	1.0 (6)
N3A—Cu1A—O3A—C19A	38.77 (19)	C13A—C14A—C15A—C16A	−1.1 (7)
N3A—Cu1A—O3A—C34A	−174.2 (4)	C14A—C15A—C16A—C11A	0.4 (7)
O1A—Cu1A—O4A—C25A	65.7 (3)	C23A—C18A—C19A—O3A	−179.3 (3)
O2A—Cu1A—O4A—C25A	−176.0 (3)	N3A—C18A—C19A—O3A	−0.2 (4)
O3A—Cu1A—O4A—C25A	−83.7 (3)	N3A—C18A—C19A—C20A	178.3 (3)
N3A—Cu1A—O4A—C25A	−21.3 (3)	C19A—C18A—C23A—C22A	0.1 (5)
O1A—Cu1A—N1A—N2A	155.7 (3)	C23A—C18A—C19A—C20A	−0.9 (5)
O1A—Cu1A—N1A—C1A	−34.3 (2)	N3A—C18A—C23A—C22A	−179.0 (3)
O2A—Cu1A—N1A—N2A	35.0 (3)	O3A—C19A—C20A—C21A	178.7 (4)
O2A—Cu1A—N1A—C1A	−154.9 (2)	C18A—C19A—C20A—C21A	0.4 (6)
O3A—Cu1A—N1A—N2A	−55.6 (3)	C19A—C20A—C21A—C22A	0.8 (7)
O3A—Cu1A—N1A—C1A	114.5 (2)	C20A—C21A—C22A—C23A	−1.5 (7)
N3A—Cu1A—N1A—N2A	−119.7 (3)	C21A—C22A—C23A—C18A	1.1 (6)
N3A—Cu1A—N1A—C1A	50.4 (2)	C25A—C24A—C29A—C28A	0.6 (5)
O1A—Cu1A—N3A—N4A	−70.8 (3)	N4A—C24A—C25A—O4A	8.6 (5)
O1A—Cu1A—N3A—C18A	107.3 (2)	N4A—C24A—C25A—C26A	−169.3 (3)
O2A—Cu1A—N3A—N4A	120.1 (3)	C29A—C24A—C25A—O4A	178.8 (3)
O2A—Cu1A—N3A—C18A	−61.8 (4)	C29A—C24A—C25A—C26A	1.0 (5)
O3A—Cu1A—N3A—N4A	138.6 (3)	N4A—C24A—C29A—C28A	171.8 (3)
O3A—Cu1A—N3A—C18A	−43.4 (2)	N4A—C24A—C29A—C30A	−7.8 (4)
O4A—Cu1A—N3A—N4A	21.7 (3)	C25A—C24A—C29A—C30A	−179.1 (3)
O4A—Cu1A—N3A—C18A	−160.2 (2)	C24A—C25A—C26A—C27A	−1.5 (5)
N1A—Cu1A—N3A—N4A	−140.8 (3)	O4A—C25A—C26A—C27A	−179.5 (3)
N1A—Cu1A—N3A—C18A	37.3 (2)	C25A—C26A—C27A—C28A	0.5 (5)
N3B—Cu1B—O4B—C25B	−29.2 (3)	C26A—C27A—C28A—C33A	−179.3 (3)
O1B—Cu1B—N1B—N2B	148.5 (3)	C26A—C27A—C28A—C29A	1.2 (5)
O1B—Cu1B—N1B—C1B	−38.8 (2)	C27A—C28A—C29A—C24A	−1.6 (5)
O2B—Cu1B—N1B—N2B	22.5 (3)	C33A—C28A—C29A—C30A	−1.6 (5)
O2B—Cu1B—N1B—C1B	−164.8 (2)	C27A—C28A—C29A—C30A	178.0 (3)
O3B—Cu1B—N1B—N2B	−67.3 (3)	C33A—C28A—C29A—C24A	178.8 (3)
O3B—Cu1B—N1B—C1B	105.5 (2)	C27A—C28A—C33A—C32A	−178.4 (3)
O4B—Cu1B—N1B—N2B	120.1 (3)	C29A—C28A—C33A—C32A	1.3 (5)
O4B—Cu1B—N1B—C1B	−67.2 (4)	C28A—C29A—C30A—C31A	1.0 (5)
N3B—Cu1B—N1B—N2B	−135.2 (3)	C24A—C29A—C30A—C31A	−179.4 (3)
N3B—Cu1B—N1B—C1B	37.5 (2)	C29A—C30A—C31A—C32A	0.0 (5)
O1B—Cu1B—N3B—N4B	−59.9 (3)	C30A—C31A—C32A—C33A	−0.4 (5)
O1B—Cu1B—N3B—C18B	111.0 (3)	C31A—C32A—C33A—C28A	−0.3 (5)
O2B—Cu1B—N3B—N4B	127.6 (3)	Cu1B—N1B—N2B—C7B	−6.9 (4)
O2B—Cu1B—N3B—C18B	−61.5 (5)	C1B—N1B—N2B—C7B	−179.9 (3)
O3B—Cu1B—N3B—N4B	148.1 (3)	Cu1B—N1B—C1B—C2B	40.6 (4)
O3B—Cu1B—N3B—C18B	−41.0 (3)	Cu1B—N1B—C1B—C6B	−137.3 (3)
O4B—Cu1B—N3B—N4B	31.3 (3)	N2B—N1B—C1B—C2B	−145.6 (3)
O4B—Cu1B—N3B—C18B	−157.7 (3)	N2B—N1B—C1B—C6B	36.5 (5)
N1B—Cu1B—N3B—N4B	−125.8 (3)	N1B—N2B—C7B—C8B	−10.9 (5)
N1B—Cu1B—N3B—C18B	45.2 (3)	N1B—N2B—C7B—C12B	173.5 (3)
O2B—Cu1B—O3B—C19B	−149.0 (2)	Cu1B—N3B—N4B—C24B	−15.2 (5)
O2B—Cu1B—O3B—C34B	6.3 (5)	C18B—N3B—N4B—C24B	173.7 (3)
O4B—Cu1B—O3B—C19B	118.0 (2)	Cu1B—N3B—C18B—C19B	41.6 (4)
O4B—Cu1B—O3B—C34B	−86.7 (5)	Cu1B—N3B—C18B—C23B	−135.6 (3)
N1B—Cu1B—O3B—C19B	−58.8 (2)	N4B—N3B—C18B—C19B	−146.3 (4)
N1B—Cu1B—O3B—C34B	96.5 (5)	N4B—N3B—C18B—C23B	36.5 (5)
N3B—Cu1B—O3B—C19B	38.9 (2)	N3B—N4B—C24B—C25B	−12.8 (5)
N3B—Cu1B—O3B—C34B	−165.7 (5)	N3B—N4B—C24B—C29B	175.9 (3)
O1B—Cu1B—O4B—C25B	50.8 (3)	N1B—C1B—C2B—O1B	2.0 (5)
O2B—Cu1B—O4B—C25B	174.0 (3)	N1B—C1B—C2B—C3B	−176.2 (3)
O3B—Cu1B—O4B—C25B	−95.0 (3)	C6B—C1B—C2B—O1B	180.0 (3)
N1B—Cu1B—O4B—C25B	76.9 (4)	C6B—C1B—C2B—C3B	1.8 (6)
N1B—Cu1B—O2B—C8B	−29.3 (3)	N1B—C1B—C6B—C5B	177.5 (4)
N3B—Cu1B—O2B—C8B	78.6 (4)	C2B—C1B—C6B—C5B	−0.3 (6)
O2B—Cu1B—O1B—C2B	111.1 (2)	O1B—C2B—C3B—C4B	180.0 (4)
O2B—Cu1B—O1B—C17B	−98.9 (4)	C1B—C2B—C3B—C4B	−2.0 (6)
O3B—Cu1B—O1B—C2B	−24.7 (3)	C2B—C3B—C4B—C5B	0.8 (6)
O3B—Cu1B—O1B—C17B	125.4 (4)	C3B—C4B—C5B—C6B	0.6 (6)
O4B—Cu1B—O1B—C2B	−154.8 (2)	C4B—C5B—C6B—C1B	−0.9 (6)
O4B—Cu1B—O1B—C17B	−4.7 (4)	N2B—C7B—C8B—O2B	1.9 (5)
N1B—Cu1B—O1B—C2B	36.3 (2)	N2B—C7B—C8B—C9B	−177.0 (3)
N1B—Cu1B—O1B—C17B	−173.7 (4)	C12B—C7B—C8B—O2B	177.2 (3)
N3B—Cu1B—O1B—C2B	−65.4 (2)	C12B—C7B—C8B—C9B	−1.6 (5)
N3B—Cu1B—O1B—C17B	84.7 (4)	N2B—C7B—C12B—C11B	178.1 (3)
O1B—Cu1B—O2B—C8B	−92.6 (3)	N2B—C7B—C12B—C13B	−2.5 (4)
O3B—Cu1B—O2B—C8B	59.6 (3)	C8B—C7B—C12B—C11B	2.2 (5)
O4B—Cu1B—O2B—C8B	174.2 (3)	C8B—C7B—C12B—C13B	−178.4 (3)
O1B—Cu1B—O3B—C34B	150.5 (4)	O2B—C8B—C9B—C10B	−179.1 (4)
O1B—Cu1B—O3B—C19B	−4.8 (3)	C7B—C8B—C9B—C10B	−0.2 (6)
Cu1A—O1A—C2A—C3A	155.8 (3)	C8B—C9B—C10B—C11B	1.3 (7)
Cu1A—O1A—C2A—C1A	−25.1 (3)	C9B—C10B—C11B—C12B	−0.6 (6)
C17A—O1A—C2A—C3A	−9.1 (5)	C9B—C10B—C11B—C16B	179.0 (4)
C17A—O1A—C2A—C1A	170.0 (3)	C10B—C11B—C12B—C7B	−1.1 (5)
Cu1A—O2A—C8A—C9A	−150.7 (3)	C10B—C11B—C12B—C13B	179.5 (3)
Cu1A—O2A—C8A—C7A	30.0 (4)	C16B—C11B—C12B—C7B	179.2 (3)
Cu1A—O3A—C19A—C18A	−27.6 (3)	C16B—C11B—C12B—C13B	−0.2 (5)
C34A—O3A—C19A—C18A	177.9 (3)	C10B—C11B—C16B—C15B	178.7 (4)
C34A—O3A—C19A—C20A	−0.4 (5)	C12B—C11B—C16B—C15B	−1.6 (6)
Cu1A—O3A—C19A—C20A	154.0 (3)	C7B—C12B—C13B—C14B	−177.9 (3)
Cu1A—O4A—C25A—C26A	−170.3 (2)	C11B—C12B—C13B—C14B	1.5 (5)
Cu1A—O4A—C25A—C24A	11.9 (5)	C12B—C13B—C14B—C15B	−1.1 (5)
Cu1A—N1A—N2A—C7A	−13.4 (4)	C13B—C14B—C15B—C16B	−0.7 (6)
C1A—N1A—N2A—C7A	176.2 (3)	C14B—C15B—C16B—C11B	2.0 (7)
Cu1A—N1A—C1A—C6A	−145.0 (3)	N3B—C18B—C19B—O3B	3.2 (5)
N2A—N1A—C1A—C2A	−154.0 (3)	N3B—C18B—C19B—C20B	−176.3 (4)
Cu1A—N1A—C1A—C2A	34.7 (4)	C23B—C18B—C19B—O3B	−179.5 (4)
N2A—N1A—C1A—C6A	26.3 (4)	C23B—C18B—C19B—C20B	1.1 (6)
N1A—N2A—C7A—C8A	−14.5 (5)	N3B—C18B—C23B—C22B	176.1 (4)
N1A—N2A—C7A—C12A	171.1 (3)	C19B—C18B—C23B—C22B	−1.0 (6)
Cu1A—N3A—C18A—C23A	−133.2 (3)	O3B—C19B—C20B—C21B	−180.0 (5)
N4A—N3A—C18A—C19A	−133.9 (3)	C18B—C19B—C20B—C21B	−0.6 (7)
Cu1A—N3A—C18A—C19A	47.7 (4)	C19B—C20B—C21B—C22B	0.2 (8)
C18A—N3A—N4A—C24A	171.6 (3)	C20B—C21B—C22B—C23B	−0.2 (7)
N4A—N3A—C18A—C23A	45.2 (4)	C21B—C22B—C23B—C18B	0.6 (7)
Cu1A—N3A—N4A—C24A	−10.2 (4)	N4B—C24B—C25B—O4B	13.0 (6)
N3A—N4A—C24A—C25A	−9.3 (5)	N4B—C24B—C25B—C26B	−164.4 (3)
N3A—N4A—C24A—C29A	−180.0 (3)	C29B—C24B—C25B—O4B	−176.1 (3)
N1A—C1A—C6A—C5A	178.3 (3)	C29B—C24B—C25B—C26B	6.4 (5)
C2A—C1A—C6A—C5A	−1.3 (5)	N4B—C24B—C29B—C28B	165.5 (3)
C6A—C1A—C2A—O1A	−178.4 (3)	N4B—C24B—C29B—C30B	−14.2 (5)
N1A—C1A—C2A—O1A	2.0 (4)	C25B—C24B—C29B—C28B	−6.3 (5)
N1A—C1A—C2A—C3A	−178.9 (3)	C25B—C24B—C29B—C30B	174.1 (3)
C6A—C1A—C2A—C3A	0.7 (5)	O4B—C25B—C26B—C27B	−180.0 (4)
C17B—O1B—C2B—C1B	174.3 (3)	C24B—C25B—C26B—C27B	−2.3 (6)
Cu1B—O1B—C2B—C1B	−27.4 (3)	C25B—C26B—C27B—C28B	−2.2 (6)
Cu1B—O1B—C2B—C3B	150.7 (3)	C26B—C27B—C28B—C29B	2.4 (5)
C17B—O1B—C2B—C3B	−7.6 (5)	C26B—C27B—C28B—C33B	−177.6 (4)
O1A—C2A—C3A—C4A	179.5 (3)	C27B—C28B—C29B—C24B	1.8 (5)
C1A—C2A—C3A—C4A	0.4 (5)	C27B—C28B—C29B—C30B	−178.5 (3)
Cu1B—O2B—C8B—C7B	23.5 (5)	C33B—C28B—C29B—C24B	−178.2 (3)
Cu1B—O2B—C8B—C9B	−157.7 (3)	C33B—C28B—C29B—C30B	1.5 (5)
C2A—C3A—C4A—C5A	−1.0 (5)	C27B—C28B—C33B—C32B	178.6 (3)
C34B—O3B—C19B—C18B	168.6 (4)	C29B—C28B—C33B—C32B	−1.4 (5)
C34B—O3B—C19B—C20B	−12.1 (7)	C24B—C29B—C30B—C31B	179.3 (3)
Cu1B—O3B—C19B—C18B	−30.1 (4)	C28B—C29B—C30B—C31B	−0.4 (5)
Cu1B—O3B—C19B—C20B	149.2 (4)	C29B—C30B—C31B—C32B	−0.9 (6)
C3A—C4A—C5A—C6A	0.4 (5)	C30B—C31B—C32B—C33B	1.1 (6)
Cu1B—O4B—C25B—C24B	15.0 (5)	C31B—C32B—C33B—C28B	0.1 (5)
Cu1B—O4B—C25B—C26B	−167.5 (3)		

### Hydrogen-bond geometry (Å, º)

**Table:**

*D*—H···*A*	*D*—H	H···*A*	*D*···*A*	*D*—H···*A*
C3*B*—H3*B*···O4*A*	0.93	2.48	3.270 (5)	143
C13*A*—H13*A*···N2*A*	0.93	2.45	2.773 (5)	100
C13*B*—H13*B*···N2*B*	0.93	2.44	2.760 (4)	100
C30*A*—H30*A*···N4*A*	0.93	2.45	2.767 (4)	100
C30*B*—H30*B*···N4*B*	0.93	2.45	2.796 (4)	100

## References

[bb1] Bruker (2006). *APEX2* and *SAINT*. Bruker AXS Inc., Madison, Wisconsin, USA.

[bb2] Farrugia, L. J. (2012). *J. Appl. Cryst.* **45**, 849–854.

[bb3] Giacovazzo, C., Burla, M. C. & Cascarano, G. (1992). *Acta Cryst.* A**48**, 901–906.

[bb4] Hallas, G. & Choi, J. H. (1999). *Dyes Pigm.* **40**, 119–129.

[bb5] Ho, M. S., Natansohn, A. & Rochon, P. (1995). *Macromolecules*, **28**, 6124–6127.

[bb6] Lin, M.-L., Tsai, C.-Y., Li, C.-Y., Huang, B.-H. & Ko, B.-T. (2010). *Acta Cryst.* E**66**, m1022. 10.1107/S1600536810028916 PMC3007315 21588097

[bb7] Millington, K. R., Fincher, K. W. & King, A. L. (2007). *Solar Energy Mater. Solar Cells*, **91**, 1618–1630.

[bb8] Sharma, G. D., Suresh, P., Sharma, S. K. & Roy, M. S. (2008). *Synth. Met.* **158**, 509–515.

[bb9] Sheldrick, G. M. (2015). *Acta Cryst.* C**71**, 3–8.

[bb10] Spek, A. L. (2015). *Acta Cryst.* C**71**, 9–18. 10.1107/S2053229614024929 25567569

[bb11] Tai, W.-J., Li, C.-H., Li, C.-Y. & Ko, B.-T. (2010). *Acta Cryst.* E**66**, m1315. 10.1107/S1600536810037888 PMC2983183 21587451

