# Supplementary figures and images for: Crystal structure of bis­{1-[(E)-(2-meth­oxy­phen­yl)diazen­yl]naphthalen-2-olato-κ3 O,N 2,O′}copper(II) containing an unknown solvate
Source: Acta Crystallogr E Crystallogr Commun. 2015 Oct 31;71(Pt 11):m207–8. doi: 10.1107/S2056989015019817 (PMC4645079; doi:10.1107/S2056989015019817)

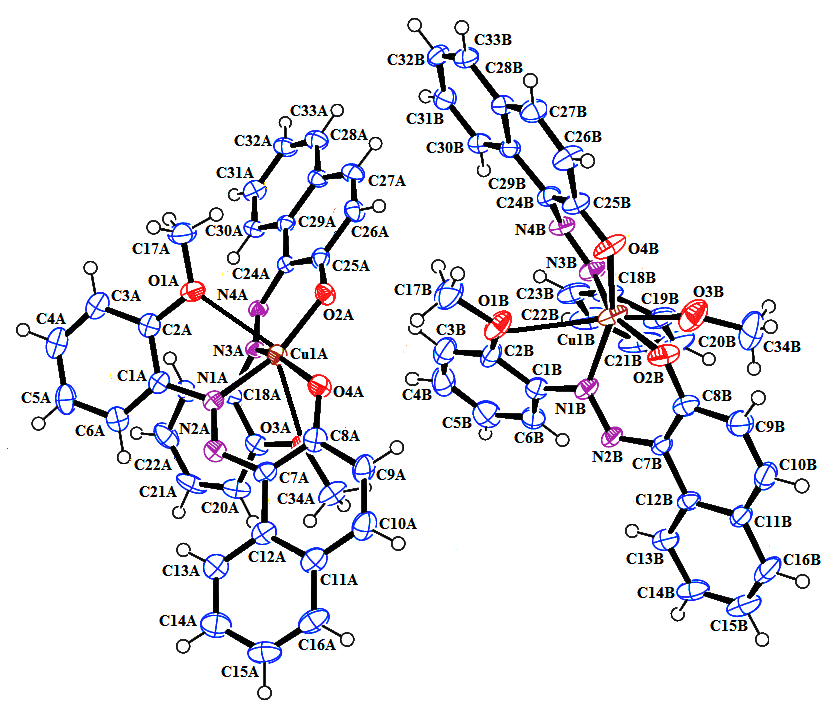

Supplement: Supplementary file 3 [file e-71-0m207-fig1.tif]

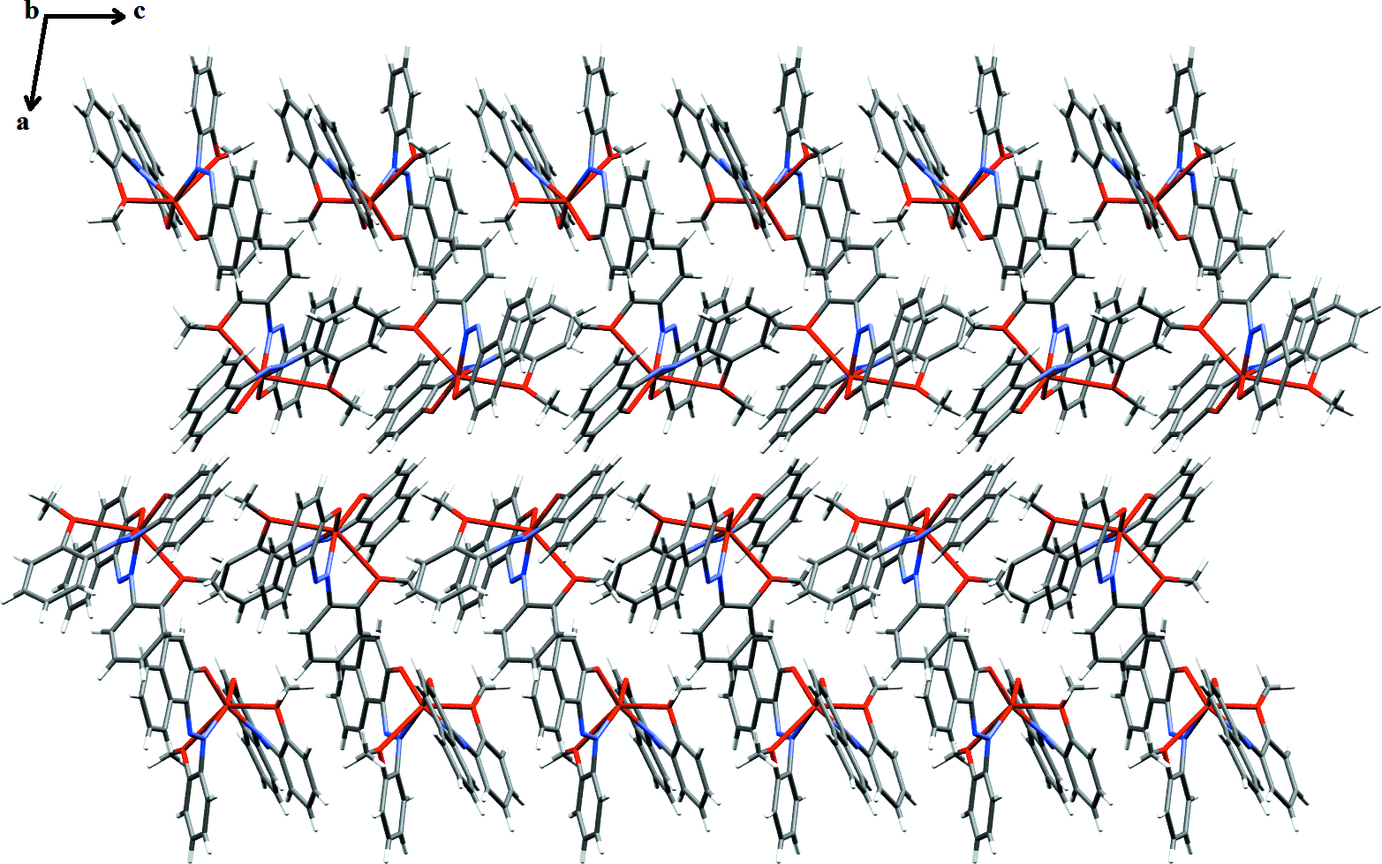

Supplement: Supplementary file 4 [file e-71-0m207-fig2.tif]
